# Endocannabinoid concentrations in major depression: effects of childhood maltreatment and relation to hippocampal volume

**DOI:** 10.1038/s41398-024-03151-z

**Published:** 2024-10-12

**Authors:** Raegan Mazurka, Kate L. Harkness, Stefanie Hassel, Niclas Stensson, Nikita Nogovitsyn, Jordan Poppenk, Jane A. Foster, Scott D. Squires, Jessie Rowe, Roumen V. Milev, Katherine E. Wynne-Edwards, Gustavo Turecki, Stephen C. Strother, Stephen R. Arnott, Raymond W. Lam, Susan Rotzinger, Sidney H. Kennedy, Benicio N. Frey, Leah M. Mayo

**Affiliations:** 1 Department of Psychiatry, Dalhousie University, Halifax, NS Canada; 2 Department of Psychology, Queen’s University, Kingston, ON Canada; 3 Department of Psychiatry, Queen’s University, Providence Care Hospital, Kingston, ON Canada; 4 Department of Psychiatry, University of Calgary, Calgary, AB Canada; 5 Hotchkiss Brain Institute, University of Calgary, Calgary, AB Canada; 6 Mathison Centre for Mental Health Reseach and Education, University of Calgary, Calgary, AB Canada; 7 Pain and Rehabilitation Centre, Department of Health, Medicine and Caring Sciences, Linköping University, Linköping, Sweden; 8 Occupational and Environmental Medicine Centre, Department of Health, Medicine and Caring Sciences, Unit of Clinical Medicine, Linköping University, Linköping, Sweden; 9 Mood Disorders Treatment and Research Centre, St. Joseph’s Healthcare, Hamilton, ON Canada; 10 Centre for Depression and Suicide Studies, St. Michael’s Hospital, Toronto, ON Canada; 11 Centre for Neuroscience Studies, Queen’s University, Kingston, ON Canada; 12 School of Computing, Queen’s University, Kingston, ON Canada; 13 Center for Depression Research and Clinical Care, Department of Psychiatry, UT Southwestern Medical Center, Dallas, TX USA; 14 Department of Comparative Biology and Experimental Medicine, University of Calgary, Calgary, AB Canada; 15 Douglas Mental Health University Institute, Department of Psychiatry, McGill University, Montreal, QC Canada; 16 Rotman Research Institute, Baycrest, Toronto, ON Canada; 17 Institute of Medical Science, University of Toronto, Toronto, ON Canada; 18 Department of Medical Biophysics, University of Toronto, Toronto, ON Canada; 19 Indoc Systems, Toronto, ON Canada; 20 Department of Psychiatry, University of British Columbia, Vancouver, BC Canada; 21 Department of Psychiatry, University of Toronto, Toronto, ON Canada; 22 Department of Psychiatry and Behavioural Neurosciences, McMaster University, Hamilton, ON Canada; 23 Center for Social and Affective Neuroscience, Department of Biomedical and Clinical Sciences, Linköping University, Linköping, Sweden

**Keywords:** Depression, Neuroscience, Biomarkers

## Abstract

Evidence from preclinical animal models suggests that the stress-buffering function of the endocannabinoid (eCB) system may help protect against stress-related reductions in hippocampal volume, as is documented in major depressive disorder (MDD). However, stress exposure may also lead to dysregulation of this system. Thus, pathways from marked stress histories, such as childhood maltreatment (CM), to smaller hippocampal volumes and MDD in humans may *depend* on dysregulated versus intact eCB functioning. We examined whether the relation between MDD and peripheral eCB concentrations would vary as a function of CM history. Further, we examined whether eCBs moderate the relation of CM/MDD and hippocampal volume. Ninety-one adults with MDD and 62 healthy comparison participants (HCs) were recruited for a study from the Canadian Biomarker Integration Network in Depression program (CAN-BIND-04). The eCBs, anandamide (AEA) and 2-arachidonylglycerol (2-AG), were assessed from blood plasma. Severe CM history was assessed retrospectively via contextual interview. MDD was associated with eCBs, though not all associations were moderated by CM or in the direction expected. Specifically, MDD was associated with higher AEA compared to HCs regardless of CM history, a difference that could be attributed to psychotropic medications. MDD was also associated with higher 2-AG, but only for participants with CM. Consistent with hypotheses, we found lower left hippocampal volume in participants with versus without CM, but only for those with lower AEA, and not moderate or high AEA. Our study presents the first evidence in humans implicating eCBs in stress-related mechanisms involving reduced hippocampal volume in MDD.

## Introduction

Major depressive disorder (MDD) is one of the most common mental illnesses and the leading cause of disability worldwide [1, 2]. Research spanning across academic disciplines and levels of analysis [3] has robustly established a causal link between stress and the onset and maintenance of MDD [4, 5]. Among all possible types of stressors, childhood maltreatment (CM) is one of the most potent risk factors for MDD [6]. Yet, even though at least half of MDD cases may be attributed to CM [6], a substantial number of those exposed to such stressors remain resilient to developing MDD or other mental health disorders [7]. Understanding the precise biological factors and processes that link CM and MDD, as well as those that confer resilience, may lead to more personalized and effective treatments, and possibly prevention efforts, for MDD.

Smaller hippocampal volume is strongly implicated as a potential biomarker of stress-related pathophysiology in MDD [8]. Importantly, research suggests that marked stress histories, such as CM, may partially account for robust findings of smaller hippocampal volume in individuals diagnosed with MDD versus healthy comparison participants [9]. However, the putative neuronal processes driving stress-related reductions in hippocampal volume in MDD may be further impacted by regulation from neuromodulatory systems such as the endocannabinoid (eCB) system.

The eCB system is comprised of receptors and ligands, which are abundant in the central nervous system and present, to a lesser degree, in the periphery. The primary endogenous ligands—N-acylethanolamine (anandamide; AEA) and 2-arachidonoylglycerol (2-AG)—demonstrate strong affinity for cannabinoid type 1 (CB_1_) receptors, which are predominantly found in the central nervous system [10]. These ligands are synthesized and secreted on demand and both are involved in dynamic regulation of the hypothalamic-pituitary-adrenal axis under basal conditions and in response to stress [11–13].

A key function of the eCB system, and in particular of AEA, is as a stress buffer [14]. For example, we have found, in humans, that higher peripheral AEA is associated with reduced negative affective responses to stress in healthy participants (i.e., both subjective and psychophysiological responses) [15, 16]. Further, preclinical research in rodent models indicates that the stress-buffering action of eCBs may extend to the neurobiological level. Specifically, Hill and colleagues found that an eCB uptake inhibitor reduced stress-induced suppression of hippocampal proliferation [17], suggesting that the eCB system may protect against processes that drive reduced hippocampal volume. Preclinical research also suggests that early-life and chronic stress exposure may lead to sustained changes in eCB regulation within the hippocampus. In humans, prolonged loss of protections against the processes driving reduced hippocampal volume could, in turn, heighten vulnerability to MDD [12, 18–20]. However, preclinical evidence implicating eCBs and hippocampal volume loss are preliminary and require translation to humans.

Among stress exposures, CM may be particularly disruptive to the eCB system since this system undergoes significant restructuring during childhood and adolescence [21]. For example, in rodent models, early life stress exposure had the most pronounced impact on the hippocampus compared to the prefrontal cortex and amygdala, resulting in downregulation of CB_1_ receptors and reductions in both AEA and 2-AG in adulthood [20]. Similarly, chronic stress exposure led to downregulation of CB_1_ receptor expression and receptor binding that was associated with reduced 2-AG content within the hippocampus, but not in the limbic forebrain [19]. Taken altogether, pathways from marked stress histories, such as CM, to smaller hippocampal volumes and MDD may *depend* on dysregulated eCB functioning. In contrast, resilience may hinge on whether regulation of this system remains intact.

Notably, studies examining peripheral eCB concentrations in MDD are mixed, with an equal number reporting significantly higher and significantly lower AEA/2-AG in MDD relative to healthy comparison groups [22–30]. These mixed findings might be clarified by considering within-group heterogeneity in MDD pathophysiology related to CM history. Specifically, support for the specific relation of CM and eCB functioning comes from a study investigating this association in individuals with and without a lifetime substance use disorder (SUD). We found that participants with CM evidenced *higher* AEA than those without CM, but only among those with *no* lifetime SUD [31]. Given its proposed role as a stress buffer, one explanation for these latter results is that innately higher levels of AEA could serve as a biological resilience factor, providing protection against developing SUD, and, possibly, other mental disorders such as MDD in the face of early marked stress exposure. In contrast, we found lower 2-AG in those with versus without a history of CM, regardless of lifetime SUD status (though post-hoc pairwise comparisons within the control group were not statistically significant). The different pattern of results for AEA versus 2-AG are in line with preclinical research indicating that the roles of AEA and 2-AG in stress processes are complementary yet distinct [12]. However, comparing the results from our study on SUD to meta-analytic evidence showing higher AEA and 2-AG across various stress-related disorders suggests that higher levels of eCBs may not necessarily signal resilience [30]. Altogether, these results underscore the need for a more nuanced approach to understanding the relationship between eCBs and stress-related disorders, taking into account the heterogeneity present within these disorders.

In the current study, our first objective was to examine whether the relation of MDD and basal eCB concentrations would vary as a function of severe CM history. We hypothesized that MDD would be associated with lower AEA concentrations compared to healthy comparison participants when MDDs also had a history of severe CM. For AEA, we further hypothesized that concentrations would be highest among participants with CM-only (i.e., without lifetime MDD). For 2-AG, we hypothesized that a current MDD diagnosis and history of severe CM would, independently, be associated with lower 2-AG concentrations.

Our second objective was to examine whether stress-related differences in hippocampal volume vary as a function of eCB concentrations. Specifically, we hypothesized that a history of severe CM and MDD would be associated with smaller hippocampal volume, but only for participants with low AEA concentrations, and not those with moderate or high AEA.

## Materials and methods

Participants were 153 adults (*n* = 91 MDD; *n* = 62 healthy comparison) recruited via community advertisements as part of a larger study on stress and reward processes from the Canadian Biomarker Integration Network in Depression program (CAN-BIND-04; see Table 1 for demographic and clinical characteristics). The study was approved by the Health Sciences Research Ethics Board at Queen’s University and Linköping Regional Ethics Review Board and carried out in accordance with the latest version of the Declaration of Helsinki. All participants provided written, informed consent.

**Table 1:** Demographic and clinical characteristics stratified by MDD status and severe CM.

	MDD (*n* = 91)		Healthy Comparison (*n* = 62)		Statistic
	Severe CM	No Severe CM	Severe CM	No Severe CM	*F* (interaction) or *X*^2^
	(*n* = 51)	(*n* = 40)	(*n* = 16)	(*n* = 46)	
Age, *M (SD)*	31.86 (15.30)	31.00 (12.54)	35.44 (18.11)	28.98 (13.52)	1.17
Sex (female), *n* (%)	39 (76.47)	28 (70.00)	10 (62.50)	33 (71.74)	0.48
Ethnicity (underrepresented racial-ethnic groups), *n* (%)	10 (20.83)	9 (22.50)	6 (40.00)	10 (22.73)	1.69
Income, *n* (%)					
High/Medium income	20 (40.00)	13 (34.21)	7 (46.67)	16 (34.781)	
Low income	14 (28.00)	7 (18.42)	2 (13.33)	4 (8.70)	
Student	16 (32.00)	18 (47.37)	6 (40.00)	26 (56.52)	
MADRS score, *M (SD)*^a^	28.82 (7.51)	26.30 (6.73)	1.44 (1.97)	1.17 (1.72)	1.24
QIDS-SR score, *M* (*SD*)^a^	16.70 (3.79)	15.62 (4.41)	3.71 (3.77)	2.95 (2.17)	0.06
AA score, *M* (*SD*)^a^	37.32 (11.79)	32.26 (11.17)	21.06 (4.04)	19.91 (3.53)	1.40
Co-occurring DSM-IV Dxs, *n* (%)^b^	34 (66.67)	24 (60.00)			0.80
Panic disorder	14	8			
Social phobia	12	13			
GAD	8	9			
Specific phobia	2	4			
Agoraphobia	1	0			
Anxiety disorder NOS	1	0			
OCD	3	1			
PTSD	11	2			
Eating disorder	2	1			
Somatoform disorder	2	0			
Number of episodes, *M (SD)*	3.22 (2.91)	2.33 (1.83)			2.75
Age of first onset, *M (SD)*	18.82 (12.87)	20.38 (9.87)			0.38
Current psychotropic medication (yes), *n* (%)	33 (64.71)	22 (55.00)			1.93

*AA* anxious arousal, *CM* childhood maltreatment, *Dxs* diagnosis, *GAD* generalized anxiety disorder, *MADRS* montgomery-åsberg depression rating scale, *MDD* major depressive disorder, *NOS* not otherwise specified, *OCD* obsessive compulsive disorder, *PTSD* posttraumatic stress disorder, *QIDS-SR* quick inventory of depressive symptomatology-self report

Low Income = total household income before taxes <$25,000; High/Medium Income = total household income before taxes >$25,000.

^a^Significant main effect of MDD status, *p* < 0.05.

^b^The frequencies do not add up to the total because some participants had more than one co-occurring diagnosis.

All participants in the MDD group met Diagnostic and Statistical Manual of Mental Disorders (DSM-IV-TR [32]) criteria for a current unipolar depressive disorder: current depressive disorder (major depressive disorder [MDD; *n* = 85], depressive disorder not otherwise specified [DNOS; *n* = 3], or dysthymia [*n* = 2]). Exclusion criteria were: lifetime bipolar disorder, psychotic disorder, alcohol or substance dependence, or medical disorder that could cause depression. Co-occurring diagnoses are reported in Table 1. Participants in the healthy comparison group had no lifetime psychiatric diagnoses. Habitual smokers, those with a neuroendocrine disorder, and women who were pregnant were also excluded [33]. See Supplementary Materials and Methods for participant flow from the initial study sample.

All participants in the current study sample had eCB and childhood maltreatment data (*n* = 153). However, due to optimization of our extraction method for AEA, 2-AG could not be detected in approximately 24% of samples, leaving a sample of *n* = 117 for these analyses. Further, of those in the initial sample, *n* = 139 completed the MRI portion of the study, all of which passed quality control. There were no significant demographic or clinical differences between participants with and without either 2-AG concentrations (*p*s > 0.072) or MRI data (*p*s > 0.056), except that participants with MRI data were significantly younger than those without (*M* = 30.2, *SD* = 13.9 and *M* = 41.0, *SD* = 16.3), *t*(151) = 2.74, *p* = 0.007.

### Measures

#### MDD diagnosis and clinical characteristics

Current and lifetime MDD and other psychiatric diagnoses were assessed using the Structured Clinical Interview for DSM-IV Axis I Disorders (SCID-I/P [34]). Clinical interviews were conducted by senior graduate students who were trained to gold-standard reliability status by KLH [35]. Severity of depression symptoms was measured with the clinician-rated Montgomery-Åsberg Depression Rating Scale (MADRS [36]) and by self-report with the Quick Inventory of Depressive Symptomatology Scale-Self Report (QIDS-SR [37]). Presence and severity of anxiety symptoms were measured with the anxious arousal (AA) subscale from the Mood and Anxiety Symptom Questionnaire (MASQ [38]). Demographic characteristics and current psychotropic medication use were assessed at the beginning of the clinical interview. We defined current psychotropic medication use as the use of one or more medications included in the neuroscience-based nomenclature system of classifying psychotropic drugs [39] (see “Supplementary Materials and Methods” for complete medication list). The decision to categorize any psychotropic medication was rooted in the understanding that eCBs play a broad role in regulating synaptic transmission and neurotransmitter release [40], suggesting possible interactions across psychotropic pharmacological classes.

#### Severe childhood maltreatment

The Childhood Experience of Care and Abuse scale (CECA [41]) is a retrospective, semi-structured, contextual interview and rating system that assesses the quality of parental care and abuse up to 18 years of age. The CECA is largely regarded as the gold-standard method for assessing childhood maltreatment [42, 43]. Presence of severe childhood maltreatment was defined as ratings of 4-marked or 3-moderate (versus 2-some or 1-little/none) on at least one of the emotional, physical, or sexual maltreatment subscales. A description of the CECA interview and rating procedures can be found in the “Supplementary Materials and Methods”.

#### Hippocampal volume

A description of MRI data acquisition procedures can be found in the “Supplementary Materials and Methods”. Automated hippocampal segmentations were performed using FreeSurfer version 7.0 (http://surfer.nmr.mgh.harvard.edu/ [44]), and a description of the image segmentation workflow [45] and our quality control procedures [46] are provided in prior reports [47]. For the purposes of the current report, we focused on total bilateral hippocampal volume to enable close comparisons between our results and those of previous studies [48–51], as well as to reduce the number of statistical comparisons. Consistent with evidence showing lateralization of hippocampal volume differences in MDD, we analyze left and right hippocampal volume, separately [52].

#### Endocannabinoid (eCB) analysis

Peripheral concentrations of the two primary eCB ligands—anandamide (AEA) and 2-arachidonylglycerol (2-AG)—were measured from blood plasma (Supplementary Materials and Methods). Analysis of the two endocannabinoid-like N-acylethanolamides (NAEs)—oleoylethanolamide (OEA) and palmitoylethanolamide (PEA)—was beyond the scope of our primary research questions, but in the interest of researchers studying NAE pathways [53], results are provided in the “Supplementary Materials and Methods”. Endocannabinoids and NAEs were analyzed using liquid chromatography tandem mass spectrometry (LC-MS/MS), as previously published [54]. Prior to analysis, outliers in eCB concentrations were winsorized to three standard deviations of the mean.

### Data analysis

We used STATA 17 for our statistical analysis and STATA or R for data visualization. We tested our first and second research questions with multiple moderated linear regression models with robust standard errors to address unequal cell sizes and heterogeneity of variance. In models testing our first research question, MDD (0- Healthy Comparison; 1-MDD), Severe CM (0 – No/Less Severe CM; 1 – Severe CM), and their interaction (MDD × Severe CM) were entered as the independent variables, with AEA or 2-AG, as the dependent variable, respectively. In models testing our second research question, MDD, Severe CM, and either of the eCBs (i.e., AEA or 2-AG), were entered as the independent variables, along with the two-way interaction terms of MDD × eCB and Severe CM × eCB, with left or right total hippocampal volume as the dependent variable, respectively. Significant interaction terms were followed-up with simple effects analyses at +/−1.5 standard deviations of the mean. We interpreted *p* values below 0.05 as statistically significant.

## Results

### Preliminary analyses for covariate selection

As expected, participants in the MDD group were significantly more likely to report a history of severe CM than those in the healthy comparison group, 56% vs. 26%, *χ*^*2*^ (1, *n* = 153) = 13.69, *p* < 0.001. Descriptive statistics of the demographic and clinical characteristics stratified by both MDD and CM are reported in Table 1. No differences in age, sex, ethnicity, or income emerged as a function of MDD status or CM. As expected, MDD participants reported significantly greater severity of depression and anxiety symptoms. Depression symptom severity (MADRS and QIDS-SR) did not differ between MDD participants with and without severe CM, *t*s < |−1.66 | , *p*s > 0.10.

Neither AEA nor 2-AG concentrations were significantly related to age, sex, ethnicity, income group, or within the MDD group, number of previous episodes, age at first onset, or depressive symptoms (MADRS; QIDS-SR; all *p*s > 0.10). However, AEA concentrations were significantly higher among MDD participants taking psychotropic medication (*M* = 0.35, *SD* = 0.17) than those not (*M* = 0.26, *SD* = 0.15), *t*(88) = −2.52, *p* = 0.013. Further, 2-AG concentrations were significantly higher among MDD participants with versus without a co-occurring disorder, *M*s = 26.51, 17.92; *SD*s = 12.66, 10.35, *t*(67) = −2.82, *p* = 0.006.

As expected, left and right hippocampal volumes were larger among men than women, *ts*(137) > | −4.53 | , *p*s < 0.001. Left and right hippocampal volume did not vary as a function of age, ethnicity, or income, or within the MDD group, by number of previous episodes, age at first onset, or depressive symptoms (MADRS; QIDS-SR), psychotropic medication use, or co-occurring diagnosis, all *p*s > 0.10.

Unless otherwise reported, including model-relevant covariates of psychotropic medication, co-occurring diagnosis, depression symptoms (MADRS or QIDS-SR), sex, and/or age in sensitivity analyses did not change the pattern of findings; therefore, we present the uncontrolled models for ease of interpretability.

### Relations of severe CM and MDD to eCBs

#### AEA

**Fig. 1:**
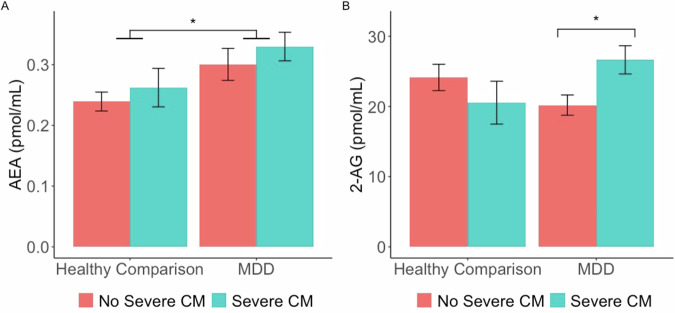
Endocannabinoid concentrations as a function of MDD and severe CM status. AEA concentrations were higher among participants with a major depressive disorder (MDD) versus healthy comparison participants (**A**). However, these results may be explained by psychotropic medication use among MDD participants (see in-text). 2-AG concentrations were higher among MDD participants, but only for those with versus without a history of severe childhood maltreatment (CM) (**B**). *p < 0.05.

The overall model predicting AEA concentrations was significant, *R*^2^ = 0.06, *F*(3, 149) = 3.91, *p* = 0.010. AEA concentrations were significantly higher in the MDD group compared to healthy comparison participants (see top panel of Table 2). Neither severe CM nor the interaction between severe CM and MDD were statistically significant (see Fig. 1A).

**Table 2:** Regression coefficients for relation of severe CM, MDD, and eCB.

*DV: AEA (n* = *153)*						
	*B*	robust SE	*t*	*p*	CI95	η^2^
CM	0.02	0.03	0.66	0.510	−0.05, 0.09	<0.01
MDD	0.06	0.03	1.99	0.048*	<0.01, 0.12	0.04
*CM* × MDD	0.01	0.05	0.13	0.896	−0.09, 0.10	<0.01
***DV: 2-AG (n*** = ***117)***						
CM	−3.59	3.96	−0.91	0.365	−11.43, 4.24	<0.01
MDD	−3.94	2.70	−1.46	0.147	−9.29, 1.40	<0.01
*CM* × MDD	10.05	4.88	2.06	0.042*	0.37, 19.72	0.03

*DV* dependent variable, *CI95* 95% confidence interval, *CM* severe childhood maltreatment, *MDD* major depressive disorder, *SE* robust standard error

*p < 0.05

Based on preliminary analyses, we conducted sensitivity analyses comparing AEA concentrations across three groups: MDD participants taking psychotropic medication (*n* = 55), MDD participants who were psychotropic medication-free (*n* = 36), and healthy comparison participants (*n* = 62). Notably, there were no significant differences between MDD participants with and without medication on any demographic or clinical characteristics (see Supplement Table 1 in Supplementary Materials and Methods). AEA concentrations were significantly higher among MDD participants taking psychotropic medications than medication-free participants with MDD, *t*(149) = 2.86, *p* = 0.013, and than healthy comparison participants, *t*(140) = 3.93, *p* < 0.001. AEA concentrations did not differ between MDD participants who were medication-free and healthy comparison participants, *t*(149) = 0.50, *p* = 0.870.

#### 2-AG

The overall model predicting 2-AG was not statistically significant, *R*^2^ = 0.05, *F*(3, 113) = 2.01, *p* = 0.117. However, the interaction between severe CM and MDD was significant (see bottom panel of Table 2). 2-AG concentrations were significantly higher among MDD participants with severe CM than MDD participants with no severe CM, *t* = 2.25, *p* = 0.026 (95% CI 0.77, 12.13; see Fig. 1B). In contrast, among healthy comparison participants, 2-AG concentrations did not significantly differ between participants with and without severe CM, *t* = −0.091, *p* = 0.365 (95% CI −11.43, 4.24). Further, neither the comparison of MDD versus healthy comparison participants was significant for those with or without severe CM, *ts* < |1.50 | , *ps* > 0.137.

We refrained from conducting sensitivity analyses on co-occurring diagnosis and 2-AG concentrations due to the small cell sizes imposed by the interaction of the three diagnostic groups (MDD participants with a co-occurring diagnosis, MDD participants without a co-occurring diagnosis, and healthy comparison participants) with the additional factor of severe CM.

### Relation of severe CM and MDD to hippocampal volume: moderation by eCBs

#### AEA

**Fig. 2:**
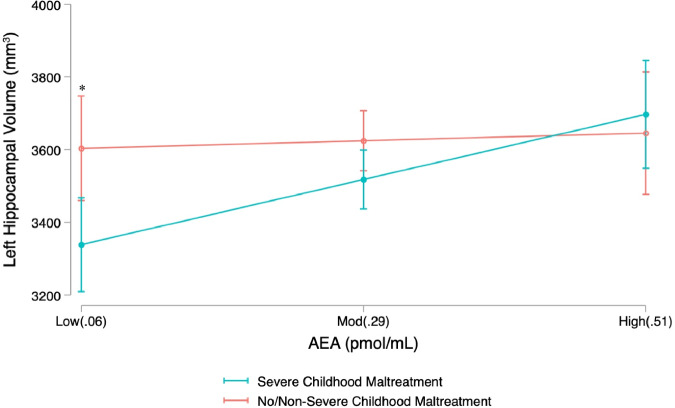
Relation of severe CM and AEA to left hippocampal volume. Hippocampal volume was smaller in participants with a history of severe childhood maltreatment (CM) compared to those without severe CM, but only among participants with low AEA, and not moderate or high AEA. Note. Low, mod, and high values represent +/− 1.5 standard deviations of the mean AEA values. *p < 0.05.

The model predicting left hippocampal volume was significant, *R*^2^ = 0.06, *F*(5, 133) = 2.77, *p* = 0.021. Neither the main effect of MDD status, AEA, or their interaction were statistically significant. However, the main effect of severe CM was statistically significant, such that a history of severe CM was associated with lower hippocampal volume (see top panel of Table 3). This main effect was further qualified by the interaction of severe CM and AEA concentrations. Follow-up simple effects analyses revealed that severe CM history was significantly associated with *lower* hippocampal volume among those whose AEA concentrations were low (1.5 standard deviations below the mean), *t* = −3.08, *p* = 0.002 (95% CI −375.14, −81.96; Fig. 2). However, there was no evidence of a significant association between CM history and left hippocampal volume among those whose AEA concentrations were at average, *t* = −1.62, *p* = 0.109 (95% CI −159.28, 16.06), or high (1.5 standard deviations above the mean), *t* = 1.16, *p* = 0.248 (95% CI −60.22, 230.87).^1^ This pattern of results was robust within the MDD group when controlling for psychotropic medication status.

**Table 3:** Regression coefficients for relation of severe CM, MDD, AEA and hippocampal volume.

DV: Left hippocampal volume (*n* = 139)						
	*B*	robust SE	*t*	*p*	CI95	η^2^
CM	−328.61	115.44	−2.85	0.005*	−556.94, −100.28	0.04
MDD	67.86	127.32	0.53	0.595	−183.97, 319.69	<0.01
AEA	289.83	409.17	0.71	0.480	−519.50, 1099.16	0.03
CM × AEA	859.39	347.49	2.47	0.015*	172.08, 1546.71	0.03
MDD × AEA	−465.31	411.42	−1.13	0.260	−1279.08, 348.47	<0.01
**DV: Right Hippocampal Volume (*n*** = **139)**						
CM	−154.59	97.83	−1.58	0.116	−348.09, 38.91	0.01
MDD	2.51	122.59	0.02	0.984	−239.98, 244.99	<0.01
AEA	527.63	467.64	1.13	0.261	−397.35, 1452.61	0.04
CM × AEA	471.65	266.40	1.77	0.079	−55.28, 998.58	0.01
MDD × AEA	−467.86	425.08	−1.10	0.273	−1308.66, 372.94	<0.01

*DV* dependent variable, *CI95* 95% confidence interval, *CM* severe childhood maltreatment, *MDD* major depressive disorder, *SE* robust standard error

*p < 0.05

The overall model predicting right hippocampal volume was significant, *R*^2^ = 0.07, *F*(5, 133) = 2.96, *p* = 0.015. However, none of the main effects of severe CM, MDD status, AEA, or their interaction terms were significant (see bottom panel of Table 3).

#### 2-AG

Neither of the models predicting left or right hippocampal volume that included 2-AG concentration as the moderator were significant, *R*^2^ = 0.03, *F*(5, 100) = 0.90, *p* = 0.482 and *R*^2^ = 0.04, *F*(5, 100) = 0.90, *p* = 0.481. Further, none of the main effects or interactions emerged as significant, all *p*s > 0.116.

## Discussion

The current study provides novel evidence for the specific relation of current MDD and severe CM history on peripheral eCB concentrations in humans. We found that MDD was associated with higher AEA and 2-AG concentrations, but these relations were specific to those using psychotropic medications (primarily antidepressants) and those with a history of severe CM, respectively. Further, we found that the relation of severe CM and left hippocampal volume depended on differences in AEA concentrations.

Our results showing that MDD was associated with higher AEA, but only for participants taking psychotropic medications, align with preclinical research demonstrating increases in AEA and 2-AG concentrations following antidepressant drug administration [55]. Further, among depressed patients, SSRI medication use has been associated with higher concentrations of both 2-AG and AEA compared to controls, though this difference was statistically significant only for 2-AG [29] (c.f [26]). Although medication use was not a primary variable of interest in our study, we speculate that higher peripheral AEA in MDD participants taking psychotropic medications versus those who were medication-free could reflect the antidepressant mechanism of action of some of these medications [56]. Specifically, higher levels of circulating peripheral AEA may signal an enhanced capacity to buffer the effects of day-to-day stress that would otherwise serve to maintain MDD [4, 14]. Future studies tracking peripheral eCBs and symptom change in response to, and over the course of, medication treatment are needed to establish this link directly.

MDD was also associated with higher peripheral blood levels of 2-AG. However, in our study, this was only the case for MDD participants with a history of severe CM, in contrast to a previous study finding CM was associated with lower 2-AG concentrations [31]. In our sample, MDD- and CM-only participants, had the lowest 2-AG concentrations, though comparisons with either of these groups did not reach statistical significance. Prior research in animal models may help to contextualize these findings. Specifically, higher 2-AG is often found across brain regions in animal models following chronic stress [12], and is associated with the passive coping style considered to behaviourally model depression [57, 58]. Thus, the fact that higher peripheral 2-AG was specific to MDD participants with CM suggests that higher circulating 2-AG may signal the kind of adverse eCB dysregulation in the central nervous system that specifically links early stress exposure with MDD. In contrast, lower peripheral 2-AG may indicate less consequential changes in eCB regulation that are not, necessarily, relevant to causal pathways of MDD. Notably, our sample size was smaller for our analyses with 2-AG analyses due to optimization of our extraction method for AEA. This could account for failure to reach statistical significance in our models, necessitating extra caution when interpreting these results.

Although the relation of MDD and peripheral eCBs did not align with the direction we hypothesized based on a single study in substance use disorder (SUD), our findings are nonetheless consistent with our conceptual model. Specifically, we posited that accounting for CM history, as well as other factors that could affect stress reactivity such as psychotropic medication use [59], may clarify inconsistent findings of both higher and lower basal peripheral eCB concentrations in MDD [22–26]. Consequently, taken together with previous research, our results suggest that the relation of CM and peripheral eCBs may vary across different clinical populations (e.g., MDD versus SUD).

Finally, as hypothesized, we found that smaller left hippocampal volume in participants with versus without a history of severe CM emerged only among participants who also had *lower* peripheral AEA concentrations. This effect was not observed for participants with CM and moderate or high AEA, nor was it observed as a function of 2-AG concentrations. Importantly, these results were robust to sensitivity analyses controlling for psychotropic medication use in the MDD-only group. These findings are the first to corroborate, in humans, that AEA may help protect against stress-related hippocampal volume loss. That is, if AEA function is intact, it may confer resilience to stress via a buffering effect. But, if AEA is low, it may signal impaired capacity to guard against the stress processes that promote smaller hippocampal volume. Indeed, research in animal models suggests that tonic AEA may be critical to *restraining* the HPA axis [60]. Thus, it is possible that chronically low AEA, when coupled with CM exposure, may contribute to sustained glucocorticoid exposure, ultimately resulting in smaller hippocampal volumes.

In light of these results, this study further reinforces what we know about the stress buffering/restraining function of AEA in (a) animal models [17] and (b) at subjective and psychophysiological levels of analysis in humans [15, 16], and extends this to the neurobiological level in humans. Further, our results align with the aforementioned evidence implicating peripheral AEA as a more direct marker of the stress buffering function of the eCB system than 2-AG, since only the model with AEA as the moderator was significant, and not the model with 2-AG. Finally, the observation that only the model for left hippocampal volume, and not right hippocampal volume, was significant aligns with previous evidence showing lateralization of hippocampal volume differences in MDD, which are likely mediated by the left CA1 region [52]. The CA1 region, being particularly sensitive to stress-related insult, may also be sensitive to the protective effects of the eCB system. Analysis of hippocampal subfields in relation to eCB levels is an area for future studies to explore.

Results should be interpreted in the context of the following limitations. First, the cross-sectional design of this study precludes making directional and/or causal conclusions regarding the relations among CM, eCB function, and MDD. Therefore, the current results should be replicated in prospective, longitudinal designs. Second, as a retrospective, self-report measure of childhood maltreatment, the CECA may be subject to recall bias in MDD. However, this contextual interview approach to retrospective assessment has greater reliability with reports of maltreatment taken at the time of the abuse (either officially documented reports or self-report) than self-report checklists [43]. Third, assessing peripheral eCBs as an index of the eCB system neglects the complexity of this system, including regulation at the level of cannabinoid receptors and across brain regions. Further, complicating interpretation of the results of the current study is the uncertainty surrounding whether peripheral eCBs accurately mirror central levels, and thus, how comparable results are to those from animal models that measure eCBs directly in the brain (c.f [15]). Thus, an important question is whether peripheral eCBs can function as reliable biomarkers for eCB signaling in the brain [61]. In future, positron emission tomography (PET) studies could add to a fuller understanding of the impact of stress exposure on the eCB system [62]. Finally, because data for the current study come from a larger research project, data collection procedures were not optimized for endocannabinoid sampling or covariate selection, which could introduce measurement error and unaccounted confounds.

Considering these limitations, our study highlights two important needs for advancing the field: (1) inclusion of peripheral eCB measurements in preclinical models in order to facilitate drawing translational parallels and extrapolating preclinical findings to clinical contexts. Even if we are able to quantify central eCB levels in humans to an extent, peripheral measurements are the only alternative for biomarker assessment in standard clinical contexts; (2) consensus guidelines for endocannabinoid measurement [63], sampling, and data collection for covariate selection to facilitate cross-study comparisons. While we emphasize caution in interpreting the results of the current study, its strengths should not be overlooked. This study leveraged an existing, uniquely rich cross-sectional dataset to directly translate and test a key mechanism from preclinical research to clinical population. Though preliminary, these results have important implications for further exploration of endocannabinoids as biomarkers of stress-related disorders, suggesting that absolute eCB concentrations must be interpreted in the context of potent enviromarkers (i.e., childhood maltreatment) and psychiatric treatments.

As mentioned, future prospective designs can address whether the increased AEA in the medication group is indeed a consequence of medication, and if so, is accompanied by any changes in symptoms or stress coping. Other future directions include examining differences in basal eCB concentrations across the episodic course of MDD as well as tracking changes in eCBs and hippocampal volume to help further understand the impact of acute versus chronic stress processes on eCB concentrations in MDD. Though we suggest AEA concentrations could signal capacity to buffer more acute life stressors, future research is needed to test how different types of stress exposure are related to eCB regulation in MDD. For example, preclinical research suggests that AEA and 2-AG are differentially impacted by different types of stress exposures (i.e., homotypic [repeated] versus heterotypic [variable] stressor type; chronic versus acute) [12].

In summary, the present findings suggest that eCB dysregulation in MDD may be specific to individuals with marked stress histories or those using psychiatric medications that potentially influence current stress reactivity. Further, our study presents the first evidence that eCB dysregulation might impact brain structure in MDD. Consequently, these findings suggest that eCBs could influence the balance of risk versus resilience to stress-related disorders in the face of childhood maltreatment and may help to explain antidepressant treatment response.

## Supplementary information

Supplementary Materials and Methods

## Footnotes

1 To test whether our results were specific to left hippocampal volume, and not due to overall volumetric differences, we re-ran the models with the residual of left hippocampal volume, parsing out either total brain volume or total intracranial volume, as the dependent variable. Results were unchanged and, therefore, are presented with left hippocampal volume in its raw form for ease of interpretation.
