# Supplementary material for: Endocannabinoid concentrations in major depression: effects of childhood maltreatment and relation to hippocampal volume
Source: Transl Psychiatry. 2024 Oct 12;14:431. doi: 10.1038/s41398-024-03151-z (PMC11470058; doi:10.1038/s41398-024-03151-z)
Supplement: Supplementary file 1 — Supplementary Materials and Methods [file 41398_2024_3151_MOESM1_ESM.docx]

**Supplementary Materials and Methods**

**Supplementary Methods**

**Participants**

As previously reported in a prior report from the overall study,^1^ a total of 440 participants took part in an initial telephone screen. Of these, 83 declined to participate, 135 were not eligible, and 3 were eligible but dropped out before their first appointment. Of the remaining 219, 13 met one or more exclusionary criteria based on the full diagnostic interview and 32 had remitted from their depressive episode by the time of the first appointment. Additionally, 21 participants were excluded for missing data on one or more of the primary variables of interest in the study: not providing a blood sample for endocannabinoid (eCB) quantification (*n* = 15), mislabeled blood samples (*n* = 1), or no/incomplete childhood maltreatment interview (*n* = 11). There were no significant demographic differences between individuals who were included versus excluded (all *p*s > .10).

**Current Psychotropic Medication**

During the clinical interview, participants were asked to report all current medications. Current psychotropic medications for the sample included amitriptyline, aripiprazole, bupropion, citalopram, clobazam, clonazepam, clonidine, desvenlafaxine, duloxetine, escitalopram, fluoxetine, gabapentin, lamotrigine, lisdexamfetamine, lorazepam, methylphenidate, mirtazapine, olanzapine, oxazepam, paroxetine, pregabalin, quetiapine, risperidone, sertraline, temazepam, topiramate, trazodone, varenicline, venlafaxine, and zopiclone.

**Severe Childhood Maltreatment**

Childhood Experience of Care and Abuse scale (CECA ^2^) interviews were conducted by senior graduate students in clinical psychology who were trained and supervised by KLH. All interviews were audio recorded. The CECA addresses concerns of subjective recall bias when using retrospective stress assessment in several ways:^3^ Participants are queried about positive and negative experiences and are asked to provide behavioral and contextual details (e.g., frequency, chronicity, degree of injury). Childhood experiences are subsequently rated by independent judges for severity using a detailed manual of rules and standardized exemplars to anchor ratings.^2^ The following scales were rated from 1-*little/none* to 4-*marked*: (a) emotional maltreatment: hostility and/or criticism directed to the child by parents; (b) physical maltreatment: violence directed toward the child by parents, including, for example, slapping, kicking, hitting, or threatening with a weapon; and (c) sexual maltreatment: age-inappropriate or non-consensual sexual activity by any perpetrator. ‘Severe childhood maltreatment’ was defined as the presence versus absence of any of the above types of maltreatment that received a rating of 3-*moderate* or 4-*marked*.

**Hippocampal Volume**

***MRI Data Acquisition.*** All neuroimaging data were collected on a single scanner at Queen’s University in Kingston, Ontario, Canada, using a Siemens 3.0 T MR Trim Trio Scanner. Whole-brain T_1_- weighted turbo-gradient echo sequences were acquired at 1 mm^3^ resolution. Pulse sequence parameters included: repetition time (TR) = 17600–1900 ms; echo time (TE) = 2.2–2.7 ms; flip 15°; inversion time (TI) = 900–950 ms; field of view 256 mm; matrix dimensions 220 × 220 and 256 × 256; contiguous slices at 1mm thickness. A vitamin E pill was used as a stereotactic marker and placed on the right side of the participant’s head. CAN-BIND neuroimaging acquisition, quality control and quality assurance protocols have been described in detail.^4^

**Endocannabinoid (eCB) Sampling**

We followed the CAN-BIND Standard Operating Procedures manual for biospecimen collection, processing, and shipping. In short, non-fasting blood samples were collected in EDTA Vacutainer Tubes by a trained phlebotomist and immediately transferred to cold storage. Plasma samples were sent to author GT’s lab at the Douglas Hospital Research Centre for storage prior to shipping to author NS’s lab at Linköping University for eCB quantification.

**Supplementary Results**

| **Supplement Table 1. Demographic and Clinical Characteristics for MDD Participants Stratified by Medication Status** | | | |
| --- | --- | --- | --- |
|  | **MDD**  **(*n* = 91)** | | **Statistic** |
|  | **Current Psychotropic Medication** | **Medication Free** | ***t* or *X*^2^** |
|  | **(*n* = 55)** | **(*n* = 36)** |  |
| Age, *M*(*SD*) | 31.95(14.41) | 30.78(13.74) | -0.39 |
| Sex (female), *n*(%) | 40(72.72) | 27(75.00) | 0.06 |
| Ethnicity (underrepresented racial-ethnic groups), *n*(%) | 10(18.18) | 9(25.00) | 0.58 |
| Income, *n*(%) |  |  |  |
| High/Medium Income | 22(40.00) | 11(30.55) | .64 |
| Low Income | 12(21.82) | 9(25.00) |  |
| Student | 20(36.00) | 14(38.89) |  |
| Severe CM, *n*(%) | 33(60.00) | 18(50.00) | .88 |
| MADRS Score, *M(SD)*^a^ | 28.58(7.89) | 26.39(6.01) | -1.42 |
| QIDS-SR Score, *M*(*SD*)^a^ | 16.33(4.47) | 16.03(3.56) | -.034 |
| AA score, *M*(*SD*)^a^ | 35.32(11.66) | 34.78(11.99) | -0.21 |
| Co-occurring DSM-IV Dxs, *n*(%) | 38(69.09) | 19(52.78) | 2.47 |
| Number of Episodes, *M (SD)* | 2.75(2.55) | 2.97(2.51) | 0.41 |
| Age of First Onset, *M (SD)* | 19.75(12.39) | 19.05(10.64) | -0.27 |
| *Note.* AA = Anxious Arousal; CM = Childhood Maltreatment; Dxs = Diagnosis; MADRS = Montgomery-Åsberg Depression Rating Scale; MDD = Major Depressive Disorder; QIDS-SR = Quick Inventory of Depressive Symptomatology-Self Report; Low Income = total household income before taxes < $25 000; High/Medium Income = total household income before taxes > $25 000. | | | |

**Preliminary Analyses with OEA and PEA**

Neither oleoylethanolamide (OEA) nor palmitoylethanolamide (PEA) concentrations were significantly related to sex, ethnicity, income group, or within the depressed group, number of previous episodes, age at first onset, comorbid diagnosis, or depressive symptoms (MADRS; QIDS; all *p*s > .10). However, OEA concentrations were significantly higher among depressed participants taking psychotropic medication (*M* = 3.27, *SD* = 1.56) than those not (*M* = 2.49, *SD* = 0.94), *t*(88) = -2.67, *p* = .009. PEA concentrations were significantly higher among older participants, *r* = .21, *p* = .010. Including age in the model of PEA did not change the pattern of results; therefore, the uncontrolled models are presented below for ease of interpretability.

**Severe CM, MDD & NAEs**

***OEA.*** The model predicting OEA was not statistically significant, *R*^2^ = .04, *F*(3, 149) = 1.81, *p* = .147. Further, none of the main effects of severe CM, MDD, or their interaction were significant (*p*s > .328; upper panel of Supplement Table 2).

***PEA.*** The model predicting PEA was not statistically significant, *R*^2^ = .05, *F*(3, 149) = 2.41, *p* = .070. However, the main effects and interaction term of severe CM and MDD were significant (see lower panel of Supplemental Table 2). PEA concentrations were higher among healthy comparison participants with severe CM than healthy comparison participants with no severe CM, *t* = 2.02, *p* = .045 (95% CI 0.01-1.24). PEA concentrations did not significantly differ between depressed participants with and without severe CM, *t* = -0.99, *p* = .325 (95% CI -0.72-0.24).

**Severe CM, MDD, NAEs and Hippocampal Volume**

***OEA.*** Neither the model predicting left or right hippocampal volume were significant, *R*^2^ = .03, *F*(5, 133) = 0.93, *p* = .462 and *R*^2^ = .02, *F*(5, 133) = 0.61, *p* = .694, and neither were any of the main effects of MDD status, OEA, or interaction terms in the model, *p*s > .080.

***PEA.*** Neither the model predicting left or right hippocampal volume were significant, *R*^2^ = .03, *F*(5, 133) = 1.06, *p* = .384 and *R*^2^ = .02, *F*(5, 133) = 0.64, *p* = .667, and neither were any of the main effects of MDD status, PEA, or interaction terms in the model, *p*s > .111.

| **Supplement Table 2. Regression Coefficients for Relation of Severe CM, MDD, and N-acylethanolamides (NAEs)** | | | | | |
| --- | --- | --- | --- | --- | --- |
| ***DV: OEA (n = 153)*** | | | | | |
|  | ***B*** | **robust SE** | ***t*** | ***p*** | **CI95** |
| **CM** | -0.02 | 0.30 | -0.06 | .953 | -0.61, 0.58 |
| **MDD** | 0.26 | 0.27 | 0.98 | .328 | -0.27, 0.79 |
| ***CM* x MDD** | 0.32 | 0.42 | 0.75 | .452 | -0.51, 1.15 |
| ***DV: PEA (n = 153)*** | | | | | |
|  | ***B*** | **robust SE** | ***t*** | ***p*** | **CI95** |
| **CM** | 0.63 | 0.31 | 2.02 | .045* | 0.01, 1.24 |
| **MDD** | 0.59 | 0.25 | 2.32 | .022 | 0.09, 1.09 |
| ***CM* x MDD** | -0.87 | 0.39 | -2.20 | .030* | -1.65, -0.09 |
| Note: DV = dependent variable; CI95 = 95% confidence interval; SE = robust standard error; CM = severe childhood maltreatment; MDD = major depressive disorder; OEA = oleoylethanolamide; PEA = palmitoylethanolamide  **p* < .05 | | | | | |

**Supplementary References**

1 Cunningham S, Mazurka R, Wynne-Edwards KE, Milev RV, Pizzagalli DA, Kennedy S *et al.* Cortisol reactivity to stress predicts behavioral responsivity to reward moderation by sex, depression, and anhedonia. *Journal of Affective Disorders* 2021; **293**: 1–8.

2 Bifulco A, Brown GW, Harris TO. Childhood experience of care and abuse (CECA): A retrospective interview measure. *Journal of Child Psychology and Psychiatry and Allied Disciplines* 1994; **35**: 1419–1435.

3 Harkness KL, Monroe SM. The assessment and measurement of adult life stress: Basic premises, operational principles, and design requirements. *Journal of Abnormal Psychology* 2016; **125**: 727–745.

4 MacQueen GM, Hassel S, Arnott SR, Addington J, Bowie CR, Bray SL *et al.* The Canadian Biomarker Integration Network in Depression (CAN-BIND): magnetic resonance imaging protocols. *Journal of Psychiatry and Neuroscience* 2019; **44**: 223–236.
